# Louise Snowball, room #237: exhibiting a multisensory mixed-media installation at a scientific dementia conference

**DOI:** 10.3389/frdem.2026.1758361

**Published:** 2026-03-25

**Authors:** Ellen Snowball, Zoe Dempster, Inbal Itzhak, Jennifer Bethell

**Affiliations:** 1 KITE Research Institute, Toronto Rehabilitation Institute, University Health Network, Toronto, ON, Canada; 2 Canadian Consortium on Neurodegeneration in Aging (CCNA), Jewish General Hospital, Lady Davis Research Institute, Montréal, QC, Canada

**Keywords:** arts-based knowledge translation, arts-based research, dementia, lived experience, patient and public engagement

## Abstract

This article describes exhibiting an interactive art installation about dementia and long-term care at the Canadian Consortium on Neurodegeneration in Aging (CCNA) Partners Forum and Science Days (PFSD), authored from the perspectives of the artist, people with lived experience of dementia and event organizers. We outline examples of collaborating with the Engagement of People with Lived Experience of Dementia (EPLED) Advisory Group to organize the exhibit. We also report evaluation data from diverse audiences, including researchers, trainees and others, demonstrating that the artwork was a highly valued part of the scientific conference, impacting perceptions of dementia and increasing awareness of lived experience engagement in research.

## 1 Introduction

Dementia is an umbrella term describing a set of symptoms related to neurodegenerative conditions affecting the brain, including Alzheimer’s disease, vascular dementia, dementia with Lewy bodies and mixed dementia. Symptoms include memory loss, difficulties in thinking, problem-solving and language, and changes in mood and behavior. Dementia is progressive; as brain cells become damaged and eventually die, symptoms worsen and affect the person’s ability to perform everyday activities (Alzheimer Society of Canada, 2024). Dementia is highly stigmatized, characterized by labeling, stereotyping, separation, status loss, and discrimination (Link and Phelan, 2001). The stigmas associated with dementia threaten the social participation of persons living with this condition and their families and can be a barrier to care and support (Herrmann et al., 2018; Vernooij-Dassen et al., 2005; Werner et al., 2014). A recent systematic review concluded that dementia-related stigmas are widespread and yet there are few evidence-based approaches to reducing stigmatizing attitudes (Herrmann et al., 2018). Given the wide-reaching impacts on persons living with dementia and their families and the growing numbers of people living with dementia, challenging the stigmas and negative public discourse around dementia has been identified as a priority for research (Bethell et al., 2018; Public Health Agency of Canada, 2024).

Despite increasing recognition of patient (and public) engagement (Forsythe et al., 2015; Manafo et al., 2018) in research as both a human right and an important step to improving research outcomes (Bethell et al., 2018; Wilson et al., 2015; Bethell et al., 2020), research in neurodegenerative conditions continues to be disease- and cure-focused, often marginalizing the subjective experience of illness (Holmes et al., 2006; Kuhlmann et al., 2024; Wall, 2008). Researchers often do not have the lived experience of the people that they study and prioritize their own scientific theories and training (Link and Phelan, 2001) and people with lived experience are still seen as study subjects rather than potential collaborators in research. Arts-based knowledge translation has become increasingly recognized as a way to shift predominant biomedical understandings of what counts as evidence (Boydell et al., 2012). Arts-based research has the potential to express lived experience perspectives and embodied forms of knowledge, thereby creating space for oft-neglected voices in research (Boydell et al., 2012; Rieger and Schultz, 2014; Rossiter et al., 2008). Art-based approaches have shown promise in reducing stigma, changing attitudes, and improving relational care toward people with dementia (Gray, 2019; Jonas-Simpson et al., 2011; Kontos and Naglie, 2007). Using arts in participatory research enables the perspectives of diverse stakeholders to be integrated, and highlights the human dimensions of illness (Boydell et al., 2012; Rieger and Schultz, 2014).

This article describes the experience exhibiting an interactive mixed-media installation at a scientific dementia conference, from the perspectives of the artist, people with lived experience and event organizers. We outline examples of collaborating together to organize the exhibit, including by involving people with lived experience of dementia (e.g., caregivers/care partners, family and friends) from the EPLED Advisory Group. We also report evaluation data collected in-person and virtually from researcher, clinician, trainee and lived experience audiences to show that the installation was viewed as a highlight of the event, impacting perceptions and awareness of dementia.

## 2 Background/Objectives

CCNA is a pan-Canadian network of clinicians and researchers working in the areas of prevention, treatment, and quality of life of neurodegenerative diseases (Chertkow et al., 2024). CCNA researchers are supported by cross-cutting programs, which help with various aspects of their research, including Training and Capacity Building; Knowledge Mobilization; and Women, Sex, Gender and Dementia. Funded by the Alzheimer Society of Canada as part of their commitment to CCNA in Phase II (2019–2024), the EPLED program was introduced to support persons with dementia and care partners to be actively involved in CCNA research activities as collaborators - not study subjects. EPLED developed an Advisory Group composed of people with diverse experiences of dementia (i.e., people living with dementia and friends, family and caregivers) from across Canada (Snowball et al., 2022). CCNA and EPLED have worked to integrate Advisory Group members as collaborators across research initiatives, involving them in active roles, including as planning committees members and as speakers at research events.

From March 19–21, 2024, CCNA held its first in-person Partners Forum and Science Days since the COVID pandemic (2020). Prior to EPLED, the scientific conference was intended for research and partner organization audiences and people with lived experience were typically not included. This event would be the first time EPLED Advisory Group members would meet the CCNA research community in-person. In the event planning stages, CCNA organizers and EPLED Advisory Group members identified the need to prioritize lived experience stories throughout the conference agenda. They invited EPLED program manager and artist, Ellen Snowball, to exhibit an interactive art installation, created as part of a Master’s thesis exhibition at the Ontario College of Art and Design University, about her lived experience with dementia.

## 3 Exhibition

**Figure 1:**
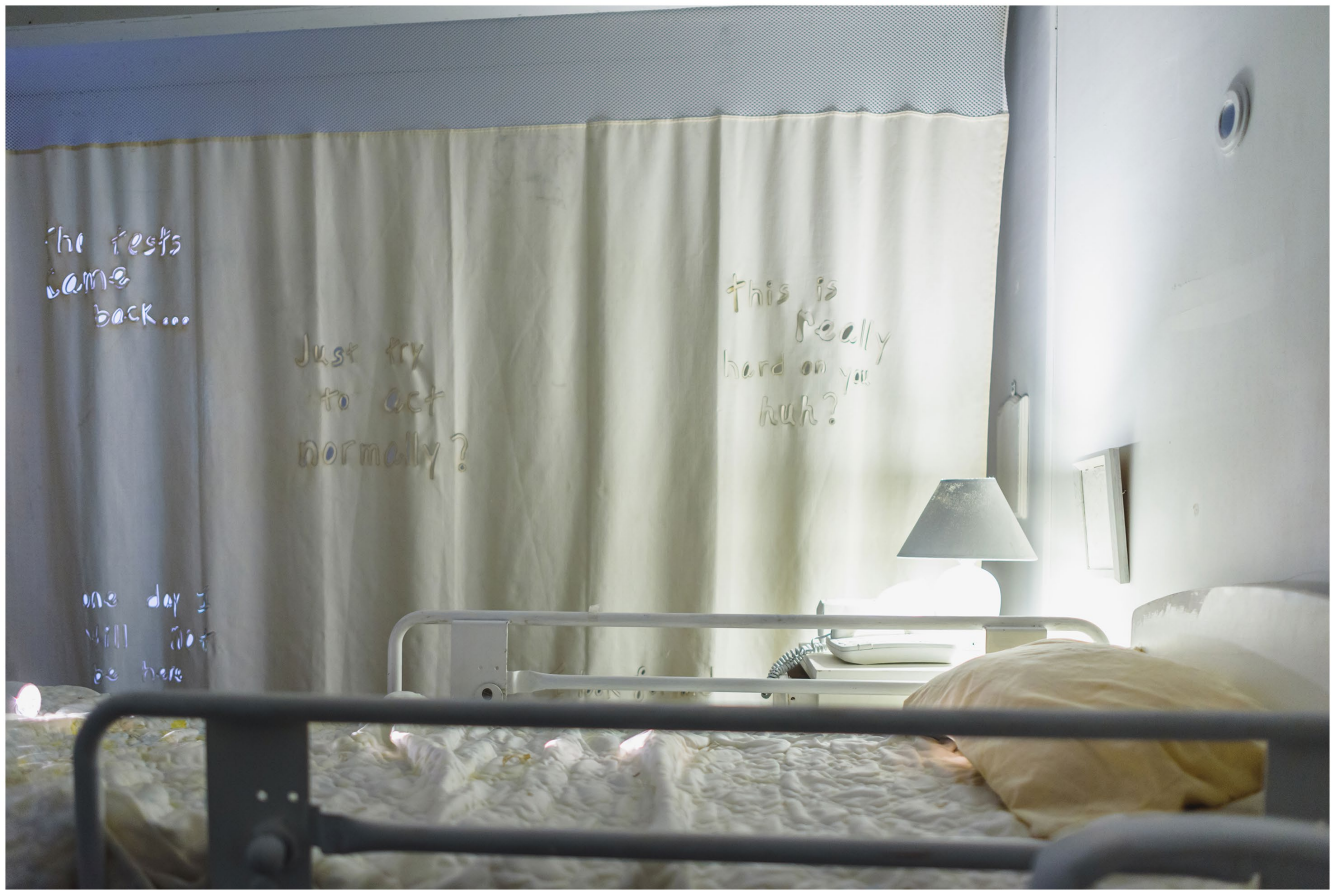
Medical bed and privacy curtain.

**Figure 2:**
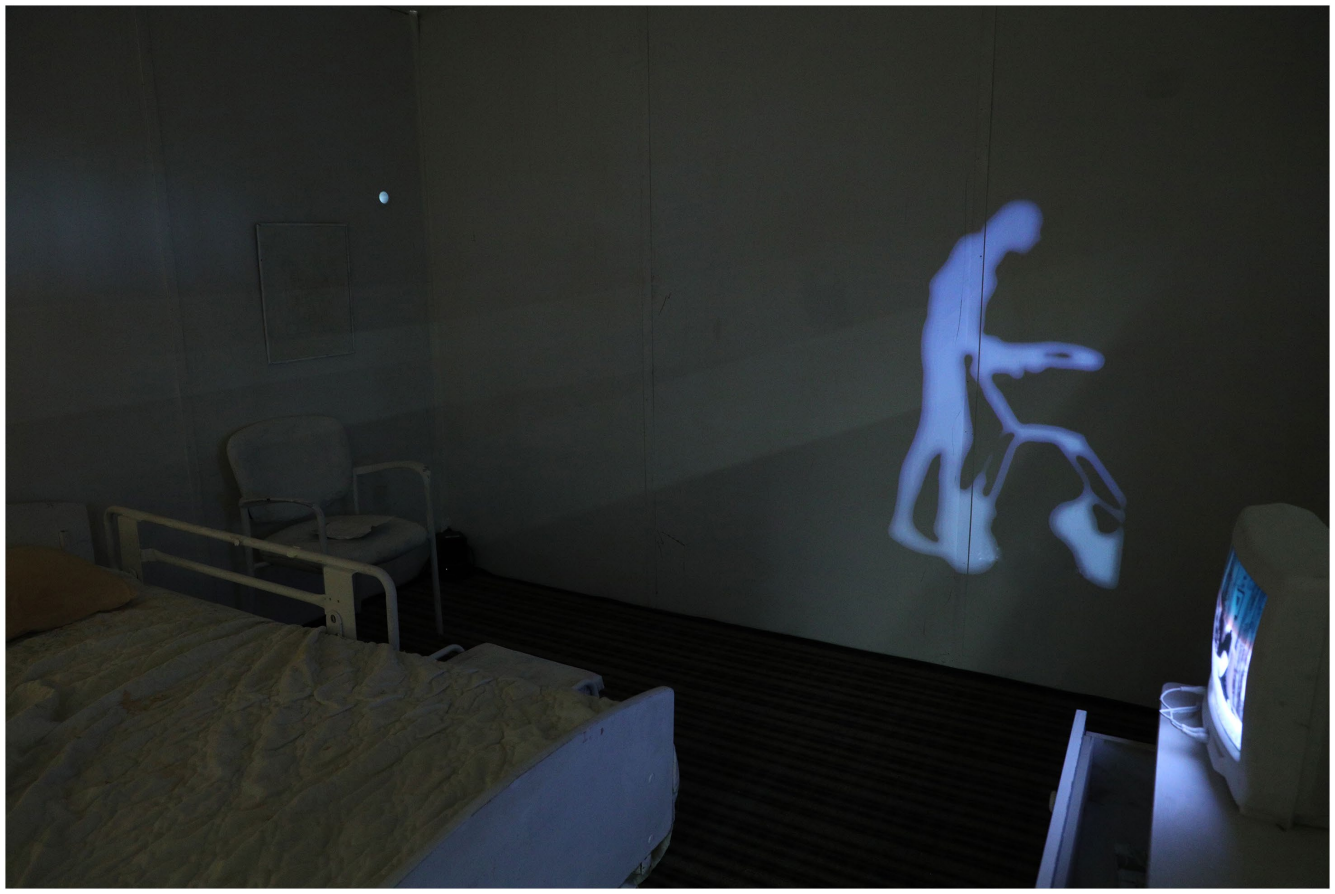
Timed projection.

Using auto-ethnography and research-creation methods, the interactive installation wove personal narratives and academic inquiry to explore personhood, identity, illness, and grief. The modular construction (8’Hx12’Wx16’L) exhibited in Montreal from March 19–21, 2024, consisted of plywood flats and soundproofing blankets (Figure 1). The window and multiple peepholes replicated The Medical Gaze, an objectifying process where people’s bodies are viewed by healthcare practitioners as separate from that of their identity (Foucault, 1963). Encouraging external visitors to peer into and observe the people that are inside, the windows caused feelings of watching/observing as well as being surveilled/observed. A sterile, white interior decorated with reused clinical objects, like a dirty privacy curtain and hospital bed, referenced The Abject - a state of rejecting what is ‘other’ to oneself (Figure 2) (Kristeva, 1982). This was further explored through a ghostly presence within the work; an audio track of Louise’s labored breathing, recorded before her death, played at a low-volume underneath a pillow on the bed.

**Figure 3:**
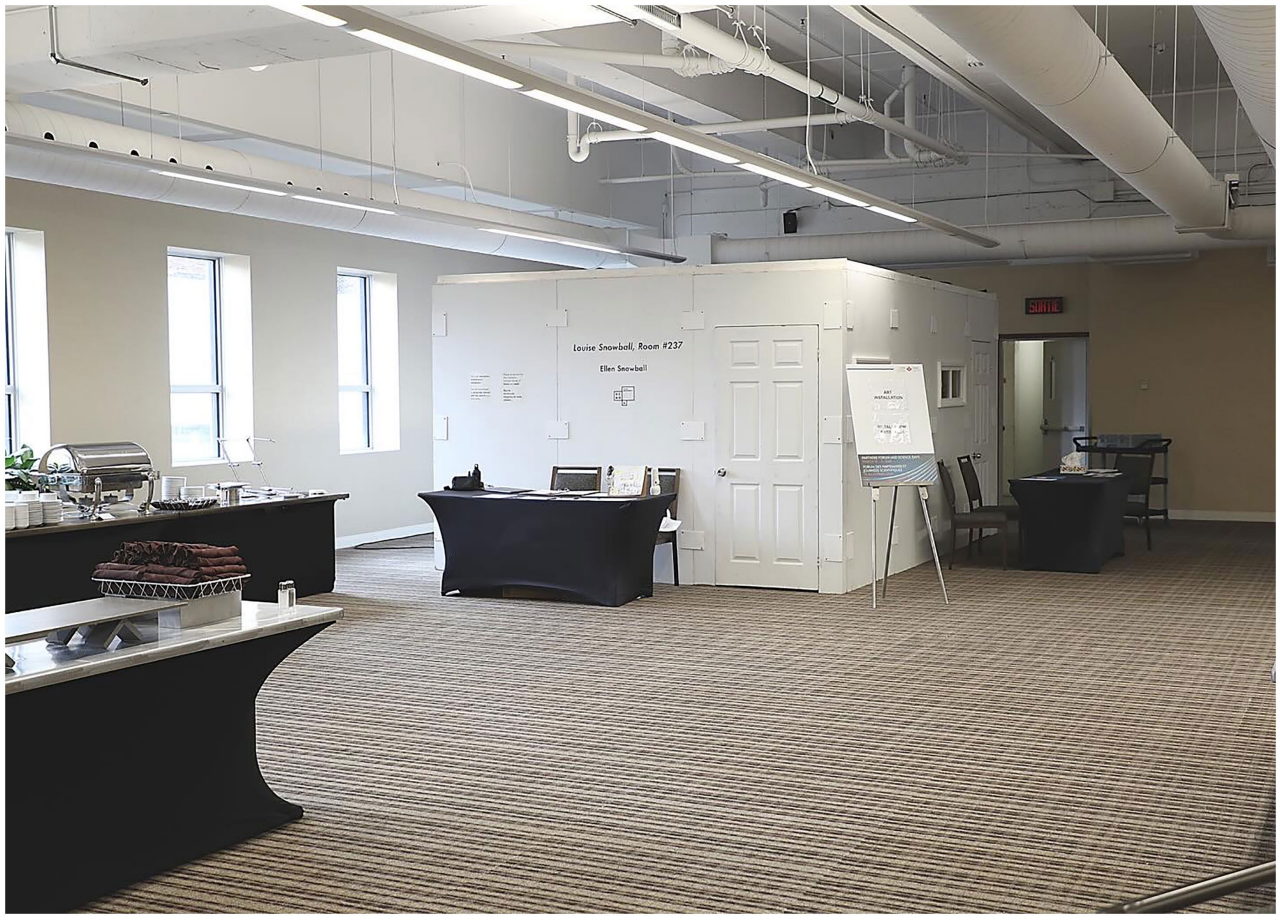
Art installation in event space.

Phenomenological themes of embodiment and selfhood were investigated through physical engagement and timed audio/visual “clues.” For instance, a projection of Louise symbolized identity beyond verbal communication, offering new knowledge through embodied experiences (Figure 3) (Merleau-Ponty, 1962). Game mechanics were used to control the environment, guiding visitors toward specific areas (Clement, 2017). Reflecting the relationship between ‘patient’ and ‘caregiver’, entry was limited to pairs of two, who could experience the work for 15-min at a time. After being equipped with flashlights, they were instructed to enter through one door and exit through another. Prompts were incorporated to elicit engagement, such as an adaptive telephone that would ring and flash intermittently. When the receiver was picked up, a vulnerable conversation between the artist and her mother could be listened to.

EPLED members, volunteering as installation monitors, asked visitors exiting the installation to fill out evaluation surveys on their experience of the artwork. The data were collected anonymously, in-person and online, using a 5-point Likert scale (rating enhanced experience of the conference, increased awareness of the value of lived experience in research and impacted perceptions of dementia) and/or via open-ended questions. EPLED Advisory Group members also handed out information sheets about the EPLED program, featuring evaluation data on their successful collaborations with researchers and program contact information.

Although a content warning was displayed outside of the installation and issued verbally to visitors, the authors observed that experiencing the installation evoked strong emotional responses in visitors. The artist, EPLED Advisory Group members and CCNA event organizers worked together to hold space for attendees, many of whom opened up about their own lived experiences of dementia. By making an emotional impact on conference attendees, we felt that the art installation facilitated important dialogues about dementia and fostered connections between researchers, trainees and people with lived experience. Evaluation data, highlighted in Table 1, corroborates that the exhibition was highly rated by different audiences for enhanced experience of the conference, increased awareness of the value of lived experience in research and impacted perceptions of dementia.

**Table 1:** Evaluation data from the art installation at Canadian consortium on neurodegeneration in aging (CCNA) partners forum and science days 2024.

Question (mean score):	Audience				Overall/total (*n =* 59)
	Trainees (*n =* 19)	Researchers (*n =* 14)	EPLED members (*n =* 9)	Other or unspecified (*n =* 17)	
To what extent did the artwork move you?	4.8	4.8	5.0	4.5	4.7
Did the artwork impact your perception about dementia?	4.7	4.2	4.7	4.0	4.4
Did the artwork enhance your experience at CCNA PFSD?	4.8	4.9	5.0	4.9	4.9
Did viewing the artwork make you think about ways PWLE can contribute to research?	4.8	4.6	5.0	4.1	4.6
Comments (example responses):	*Excellent piece. Your voice is an integral part arguably most important to research! Thanks.* - Trainee *This was a phenomenal experience - I do not even have the words to describe how much this moved me. More than any scientific paper ever could.* - Trainee *This is a wonderful way to capture the feeling and emotion beyond science presented.* - Trainee *Fantastic. It conveys the caregiver experience for any long term death.* - Researcher *These artworks absolutely enrich the experience. - Researcher* *What a journey of love. A tribute beyond words, just tears from the heart.* - EPLED member *It is especially impactful to incorporate lived experience & the interactive aspect is very refreshing in such an academic context.* - EPLED member				

## 4 Conclusion

In this article, we described exhibiting an interactive art installation about dementia at a scientific conference, from the perspectives of the artist, people with lived experience and event organizers. By bridging the gap between art, lived experience and research, we aimed to facilitate an embodied, emotional experience in audiences, fostering new perspectives on dementia’s impact and generating interest in lived experience engagement in research. Future considerations include offering mental health support to minimize potential negative impacts on audiences and monitors (e.g., retraumatization) and implementing a more developed evaluation plan (e.g., collecting demographic data, expanding objectives). We hope that this article can offer insight on how art can be used to meaningfully communicate lived experience perspectives in research contexts.

## Data Availability

The original contributions presented in the study are included in the article/supplementary material, further inquiries can be directed to the corresponding author.
