# Correction: Louise Snowball, room #237: exhibiting a multisensory mixed-media installation at a scientific dementia conference

**DOI:** 10.3389/frdem.2026.1845196

**Published:** 2026-04-20

**Authors:** Ellen Snowball, Zoe Dempster, Inbal Itzhak, Jennifer Bethell

**Affiliations:** 1 KITE Research Institute, Toronto Rehabilitation Institute, University Health Network, Toronto, ON, Canada; 2 Canadian Consortium on Neurodegeneration in Aging (CCNA), Jewish General Hospital, Lady Davis Research Institute, Montréal, QC, Canada

**Keywords:** arts-based knowledge translation, arts-based research, dementia, lived experience, patient and public engagement

There was a mistake in the caption of “Figure 1”, “Figure 2”, and “Figure 3” as published. The captions read “Figure 1: Art installation in the event space”; “Figure 2: Medical bed and privacy curtain”; Figure 3: Time projection” The corrected caption of of “Figure 1”, “Figure 2”, “Figure 3” appears below.

**Figure 1:** Medical bed and privacy curtain.

**Figure 2:** Timed projection.

**Figure 3:** Art installation in event space.

The original version of this article has been updated.

